# The Young Heart Tears Easily Apart: A Case Report of Spontaneous Coronary Artery Dissection

**DOI:** 10.7759/cureus.15590

**Published:** 2021-06-11

**Authors:** Harish Ravipati, Shelden Rodrigues, Swathi Rao, Buddhi Hatharaliyadda, Christine Junia

**Affiliations:** 1 Internal Medicine, MacNeal Hospital, Berwyn, USA; 2 Internal Medicine, Macneal Hospital, Berwyn, USA

**Keywords:** spontaneous coronary artery dissection, percutaneous coronary intervention, coronary dissection, : acute coronary syndrome, coronary artery bypass grafting(cabg), chest pain, st-elevation myocardial infarction (stemi)

## Abstract

Spontaneous coronary artery dissection (SCAD) is a rare cause of acute coronary syndrome (ACS), seen mostly in young females. The rarity and limited knowledge of the disease make its management challenging. Prompt diagnosis of the condition is extremely important to decrease both long- and short-term complications. Treatment options depend on hemodynamic stability and the location of the dissection- with more distal lesions treated more conservatively as opposed to proximal lesions which are treated with percutaneous coronary intervention (PCI) or coronary artery bypass graft (CABG). The following are the two cases with different presentation, management and outcomes.

Our first patient was a 35-year-old woman with no medical history who presented with acute, anginal pain, diaphoresis and palpitations. She was hemodynamically stable on presentation, with work-up significant for electrocardiogram (ECG) with sinus bradycardia, ST elevation in leads V1-V6, and elevated troponin level of 4 ng/ml. There was no evidence of a pulmonary embolism on computed tomography (CT) of the chest. A coronary angiogram showed 100% dissection of the proximal to mid-left anterior descending (LAD) artery. Attempts to place a stent in the proximal to mid LAD were unsuccessful as the true lumen of the LAD was not accessible. The patient became hemodynamically unstable, and an emergent CABG was done, restoring blood flow. The patient recovered during her hospital stay and was discharged with dual antiplatelet therapy (DAPT), beta-blockers, and atorvastatin.

The second patient was a 28-year-old woman, with a history of hypertension who presented with anginal chest pain. Workup showed ECG with minimal ST elevations in anteroseptal leads, with elevated troponin level to 0.71 ng/ml. Coronary angiogram showed 40-50% stenosis of the mid LAD with an aneurysmal segment. An echocardiogram showed no evidence of wall motion abnormalities, and she had a normal left ventricular ejection fraction (LVEF). She was discharged home the next day, on medical management. After two days, she returned to the hospital with similar complaints, with work-up significant for ECG with non-specific ST-T abnormality, and troponin level which peaked at 2.22 ng/ml. She was started on a heparin drip, and a repeat left heart catheterization revealed type 2 dissection of the mid to distal LAD, with intravascular ultrasound showing a fractional flow reserve of 0.76. She was discharged home on DAPT, beta-blocker, calcium channel blocker (CCB), and atorvastatin, with close cardiology follow up.

These two cases highlight the importance of keeping in mind the possibility of SCAD, especially when relatively healthy young women present with anginal symptoms. Early diagnosis of the condition and prompt management are extremely important to ensure favourable outcomes. The two cases also describe the coronary angiogram findings in SCAD, and the different strategies employed in the management of this condition.

## Introduction

SCAD is a rare non-atherosclerotic, non-traumatic, non-iatrogenic cause of ACS. SCAD usually occurs in young female patients. Because of its rarity, knowledge of SCAD is limited. Timely diagnosis is important as the management is different based on the location of the SCAD, hemodynamic status and other causes of ACS. Prompt treatment and proper counseling decrease both short- and long-term complications. We present two cases of SCAD that were managed differently.

## Case presentation

Case 1

**Figure 1:**
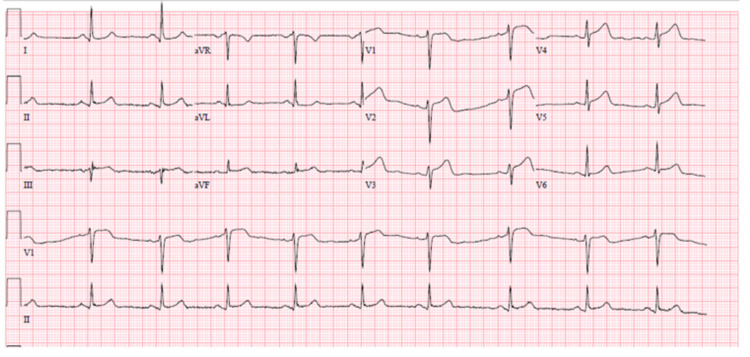
ECG on admission showing ST segment elevations in leads V1 to V6

A 35-year-old female patient with no past medical history presented with sudden onset of non-exertional, substernal, chest pain, radiating to left side of chest and left arm, associated with non-exertional dyspnoea, diaphoresis and palpitations. Past surgical history was non-significant. There was no family history of coronary artery disease, spontaneous coronary artery dissection, fibromuscular dysplasia, vascular or connective tissue disorders. Patient was an ex-smoker with five pack year smoking history, quit ten years ago, drinks three glasses of alcohol per week, and no history of recreational drug use. Patient was not on medications and had no known allergies. Vitals were stable at presentation. The physical examination was unremarkable. Labs were only significant for initial troponin level elevated to 4 ng/ml. Chest X ray was unremarkable. Initial ECG showed sinus bradycardia, ST segment elevations in leads V1 to V6 (Figure 1). No changes were noted on serial ECG’s.

Differentials included acute coronary syndrome, myocarditis, pericarditis, spontaneous coronary artery dissection, aortic dissection, and pulmonary embolism. CT of chest, abdomen and pelvis with contrast did not reveal pulmonary embolism, aortic dissection or other vascular abnormalities. As chest pain was associated with elevated troponin level and ECG changes, ACS was high on the differential; coronary angiography was performed which showed 100% mid LAD coronary artery dissection (Video 1), no significant coronary atherosclerosis was identified in the remaining vessels. Type 1 spontaneous coronary artery dissection of proximal to mid LAD coronary artery was diagnosed and as the patient became hemodynamically unstable, coronary angiogram was done to place a stent in proximal to mid LAD which was unsuccessful as the true lumen of the LAD coronary artery was not accessible on multiple attempts. Emergent CABG was done because of proximal to mid LAD coronary artery involvement and hemodynamic instability. An intraoperative trans-esophageal echo (TEE) before CABG showed 40% LVEF. Intraoperatively, dissection was noted to have started at the junction of proximal to mid LAD with severe thrombosis extending to apex. Left internal mammary artery grafting was done to proximal to mid LAD and coronary blood flow was restored. LVEF improved to 60% on intraoperative TEE, hemodynamically stabilized post CABG, and the patient was transferred to intensive care unit. Patient improved gradually during the hospital course and was discharged to home on dual antiplatelet therapy, atorvastatin and metoprolol. Patient was evaluated for extra coronary vascular abnormalities with CT angiography of head which did not reveal abnormalities. Four months later, patient was admitted to hospital with mild chest pain, concerning for recurrent SCAD. Patient was monitored in the hospital with serial troponins, ECG, which were not significant and as the suspicion was low, no coronary angiography was performed. Patient was counseled on recurrent chest pain, exercise regimen and follows as outpatient with no further complications.

**Video 1:** Coronary angiogram showing SCAD in proximal, mid and distal left anterior descending artery.

Case 2

**Figure 2:**
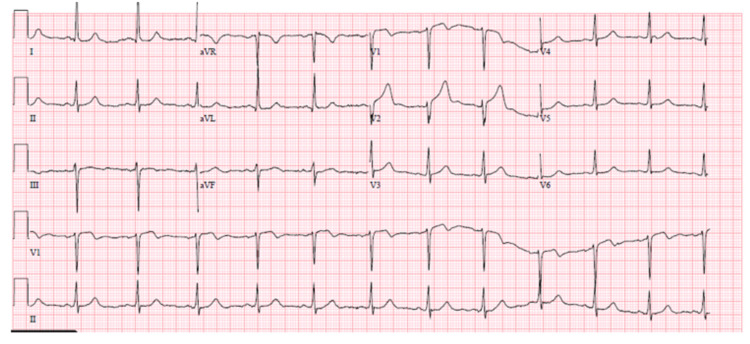
ECG during first admission showing ST-segment elevations in leads V1 and V2.

A 28-year-old female with a past medical history of hypertension and smoking presented with acute onset of substernal substernal pressure-like chest pain for two days. Patient reported pain radiating to left arm, associated with palpitations and dizziness. Patient had no past surgical history, no family history of SCAD, premature CAD, and vascular disorders. Patient smokes three cigarettes per week, socially drinks alcohol, and has no history of recreational drug use. Patient was on hydrochlorothiazide, and had no known allergies. Patient was in significant distress but had an otherwise unremarkable exam. ECG showed minimal ST elevations in V1, V2 (Figure 2).

**Figure 3:**
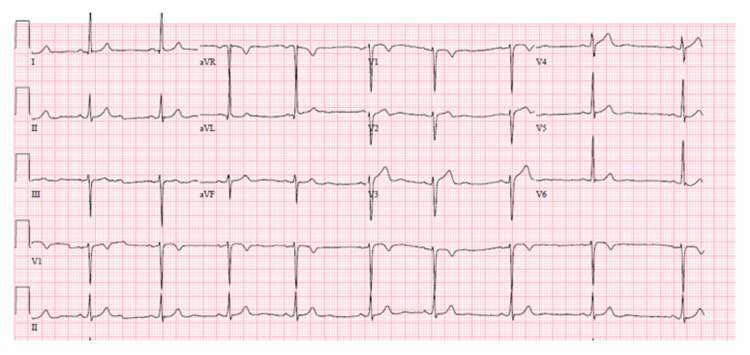
ECG during second admission showing nonspecific ST T wave abnormality.

Initial troponin I was elevated to 0.71 ng/mL. Patient was given aspirin, nitrate, beta-blocker and atorvastatin in emergency room and started on a heparin drip for ACS. Coronary angiogram showed 40-50% stenosis in the mid LAD with an aneurysmal segment and no involvement of other coronaries; stenting was hence deferred. TTE showed LVEF of 55% and no left ventricular wall motion abnormalities. Patient was discharged the next day on aspirin, beta blocker, atorvastatin, and nitrate. Two days later, she was readmitted with a similar presentation. Initial troponin level was elevated to 2.22 ng/mL. ECG showed nonspecific ST T wave abnormality (Figure 3). 

Patient was started on a heparin drip as well as a calcium-channel blocker for possible coronary vasospasm. Overnight she developed episodes of non-sustained ventricular tachycardia. Repeat left heart catheterization revealed Type 2 mid to distal left anterior descending spontaneous coronary dissection (Video 2). Her fractional flow reserve was deemed to be 0.76 with intravascular ultrasound (IVUS) and the decision to continue medical management alone was made. Patient was discharged home on DAPT, beta-blockers, calcium-channel blocker, atorvastatin, and a follow-up with cardiology. Patient did not continue care with us and presence of other vascular or connective tissue disorders could not be excluded.

**Video 2:** Coronary angiogram showing SCAD in proximal left anterior descending artery.

## Discussion

These two cases highlight the importance of early diagnosis, indications of percutaneous coronary intervention, surgical management in SCAD and counseling on post hospital symptoms. SCAD is common in young female patients with mean age of onset 47-53 years, with less common risk factors like hypertension, diabetes mellitus, and dyslipidemia compared to atherosclerotic coronary artery disease [1, 2]. Exertion-related stressors are common in males whereas emotional stressors are common in females. Pathophysiology of SCAD is dissection of tunica intima from media. Two hypotheses are proposed: “outside-in”, which is development of intramural hematoma within the vessel wall by rupture of vasa vasorum before its propagation and connection to true lumen, is considered most common, while the “inside-out” suggests that a tear happens first and then creates a false lumen between tunica intima and media.

The diagnosis is made by coronary angiography, optical computed tomography, and intravascular ultrasound. Four types of SCAD are described based on angiographic findings: Type 1 is defined as the presence of connection between true and false lumen; Type 2: most common type of SCAD; the dissection is greater than 20 mm in length with no connection between true and false lumen. Type 3 has a dissection of less than 20 mm and no connection between true and false lumen. Type 4 has a dissection and obstruction to flow at the end of the coronary arteries which mimics an embolus [1]. In case 1, coronary angiography alone was performed as true and false lumens were well visualized. In case 2, as coronary angiography was unclear, IVUS was performed to diagnose and classify SCAD. Troponins are usually elevated, and an EKG shows signs of ischemia or infraction similar to ACS which aids diagnosis, and also helps in understanding the time of onset and progression. Cardiac magnetic resonance can be used in selected inconclusive cases to improve early diagnosis and outcomes [3].

Treatment is usually conservative if the dissection is more distal and patient is hemodynamically stable with no dynamic changes on ECG [4]. Patients with SCAD due to “inside-out” with intimal dissection responds better to conservative management rather than patients with SCAD from “outside-in” mechanism. In case 2, as the dissection involves mid to distal LAD and the patient was hemodynamically stable, she was treated with medical therapy [4]. PCI or CABG is considered if the dissection is more proximal, patient is hemodynamically unstable, dynamic ST segment changes and ventricular arrhythmias. In case 1, as the dissection is more proximal and patient was hemodynamically unstable, PCI, and later CABG were done. Vein grafts are recommended over arterial grafts in young patients requiring CABG from SCAD, as arterial grafts are reserved for the later years if patients develop an atherosclerotic ischemic event that requires repeat CABG. No specific recommendations are available on the duration of antiplatelet therapy. DAPT is generally considered for the first two to three months while aspirin is continued lifelong. Anticoagulants should be avoided after diagnosis of SCAD. Beta blockers are the main stay of treatment and has mortality benefit [2]. Nitrates and calcium channel blockers are used for angina symptoms. Angiotensin converting enzyme inhibitors or angiotensin receptor blockers are not indicated for isolated SCAD. Antilipidemic drugs are only indicated in patients with dyslipidemias. Along with the regular complications of PCI and CABG, complications specific to SCAD are stent migration after PCI and loosening of sutures after CABG, after healing of dissection. Patients should be observed for at least 3-5 days before discharge to prevent readmissions from recurrent SCAD, as majority of patients readmit with in 2 days post discharge with less in hospital stay.

SCAD is one of the major causes of ACS in pregnancy [5]. Pregnancy related SCAD (P-SCAD) is defined as SCAD during pregnancy or less than 12 weeks postpartum, and occurs mostly within one to two weeks after delivery [2, 3, 5]. P-SCAD presents with more ST-T wave segment changes and multivessel disease [5]. In their systematic review on P-SCAD, Paratz et al described increasing incidence of P-SCAD in primigravidas in the recent years. A study from Mayo clinic SCAD registry by Tweet et al described that the incidence of P-SCAD is more in multiparous women overall [5, 6]. In patients with P-SCAD, there is a significant upward trend towards caesarean section with better maternal and fetal outcomes [5]. Hormonal changes are also thought to have a role because of their presentation in young females and relation to menstruation [2, 3, 7]. It is unclear if in vitro fertilization affects P-SCAD incidence due to hormonal interactions. Recurrence rates are similar between P-SCAD and SCAD in non-pregnant patients. Due to minimal radiation exposure with proper protection, P-SCAD patients should be managed similar to other SCAD patients.

Myocardial bridging is the intramyocardial part of epicardial coronary artery with a prevalence of 30 to 40%. The clinical significance of SCAD with myocardial bridging is not well understood. LAD is the most commonly affected coronary artery with SCAD due to myocardial bridging mostly in the bridging part or distal part of the artery. Tajrishi et al hypothesized mechanisms by which myocardial bridging can predispose to SCAD which includes vasospasm of the artery in the area of myocardial bridging, decreased response to vasoactive agents and increased pressure in the bridging artery leading to disturbances in the flow [8]. It is unclear if the treatment of myocardial bridging would prevent SCAD. Many endocrine disorders are frequently associated with cardiac diseases and the association of hypothyroidism with SCAD is rare. Patients with hypothyroidism showed more diffuse and distal lesions with SCAD. Camacho et al showed SCAD is more associated with hypothyroidism, more common in females and mostly managed conservatively [9].

Connective tissue diseases are commonly associated with SCAD with fibromuscular dysplasia being the most common anomaly. Additional imaging of head, chest, abdomen, and pelvis with contrast is necessary to rule out fibromuscular dysplasia, connective tissue, and other vascular disorders [1, 2]. Diagnosing associated connective tissue and vascular disorders and determining genetic sequences associated with SCAD helps in identifying patients at risk and prevent recurrent SCAD by managing stressors and appropriate genetic counseling. Genetic studies have shown the association of the SCAD with the PHACTR1/EDN1 locus. Antonutti et al described gene mutations in COL3A1, COL5A2, FBN2, LTBP2, NOTCH1 and ELN genes and their association with major adverse cardiovascular events including recurrent SCAD, cardiogenic shock and heart failure [10]. These gene mutations are associated with proximal SCAD and absence of these gene mutations are associated with more distal artery involvement [10].

Upon discharge, counseling about recurrent chest pain, exercise and pregnancy is essential to prevent readmissions, decrease recurrent SCAD and pregnancy-related adverse outcomes. Understanding hormonal effects on SCAD helps in guiding the appropriate post- SCAD contraceptive options in females. Counseling about contraception is important in patients who had SCAD and P-SCAD. The goal of contraception is to use the most effective method with low hormonal interaction.

## Conclusions

Spontaneous coronary artery dissection should be considered as a differential diagnosis in ACS, especially in young female patients with low cardiac risk factors. Physicians should be well aware of management options, educating patients on discharge about the risk factors and lifestyle modifications.
